# Overview of Circadian Rhythms

**Published:** 2001

**Authors:** Martha Hotz Vitaterna, Joseph S. Takahashi, Fred W. Turek

**Affiliations:** Martha Hotz Vitaterna, Ph.D., is a senior research associate in the Center for Functional Genomics, Northwestern University, Evanston, Illinois. Joseph S. Takahashi, Ph.D., is the director of the Center for Functional Genomics, the Walter and Mary E. Glass Professor in the Department of Neurobiology and Physiology, and an investigator at the Howard Hughes Medical Institute, Northwestern University, Evanston, Illinois. Fred W. Turek, Ph.D., is the director of the Center for Sleep and Circadian Biology and is the Charles T. and Emma H. Morrison Professor in the Department of Neurobiology and Physiology, Northwestern University, Evanston, Illinois

## Abstract

The daily light-dark cycle governs rhythmic changes in the behavior and/or physiology of most species. Studies have found that these changes are governed by a biological clock, which in mammals is located in two brain areas called the suprachiasmatic nuclei. The circadian cycles established by this clock occur throughout nature and have a period of approximately 24 hours. In addition, these circadian cycles can be synchronized to external time signals but also can persist in the absence of such signals. Studies have found that the internal clock consists of an array of genes and the protein products they encode, which regulate various physiological processes throughout the body. Disruptions of the biological rhythms can impair the health and well-being of the organism. Key words: circadian rhythm; time of day; biological regulation; biological adaptation; temperature; light; hypothalamus; neural cell; gene expression; mutagenesis; sleep disorder; physiological AODE (effects of alcohol or other drug use, abuse, and dependence)

One of the most dramatic features of the world in which we live is the cycle of day and night. Correspondingly, almost all species exhibit daily changes in their behavior and/or physiology. These daily rhythms are not simply a response to the 24-hour changes in the physical environment imposed by the earth turning on its axis but, instead, arise from a timekeeping system within the organism. This timekeeping system, or biological “clock,” allows the organism to anticipate and prepare for the changes in the physical environment that are associated with day and night, thereby ensuring that the organism will “do the right thing” at the right time of the day. The biological clock also provides internal temporal organization and ensures that internal changes take place in coordination with one another.

The synchrony of an organism with both its external and internal environments is critical to the organism’s well-being and survival; a lack of synchrony between the organism and the external environment may lead to the individual’s immediate demise. For example, if a nocturnal rodent were to venture from its burrow during broad daylight, the rodent would be exceptionally easy prey for other animals. Similarly, a lack of synchrony within the internal environment might lead to health problems in the individual, such as those associated with jet lag, shift work, and the accompanying sleep loss (e.g., impaired cognitive function, altered hormonal function, and gastrointestinal complaints).

The mechanisms underlying the biological timekeeping systems and the potential consequences of their failure are among the issues addressed by researchers in the field of chronobiology.^1^ In its broadest sense, chronobiology encompasses all research areas focusing on biological timing, including high-frequency cycles (e.g., hormone secretion occurring in distinct pulses throughout the day), daily cycles (e.g., activity and rest cycles), and monthly or annual cycles (e.g., reproductive cycles in some species). Among these interrelated areas of chronobiology, this article focuses on one frequency domain—the daily cycles known as circadian rhythms. (The term “circadian” derives from the Latin phrase “circa diem,” which means “about a day.”) Although virtually all life forms— including bacteria, fungi, plants, fruit flies, fish, mice, and humans—exhibit circadian rhythms, this review is primarily limited to the mammalian system. Other animals are discussed only in cases in which they have contributed to the understanding of the mammalian system, particularly in studies of the molecular genetic makeup of the timekeeping system. (For comparative discussions of other nonmammalian model systems that have contributed to the depth of understanding of circadian rhythmicity in mammals, the reader is referred to Wager-Smith and Kay 2000.) Overall, this article has the following major objectives: (1) to provide a highly selective historical overview of the field, (2) to review characteristic properties of circadian rhythms, (3) to define the structural components and the molecular genetic mechanisms comprising the biological clock, and (4) to explore the health effects of biological rhythms.

## Historical Overview of Chronobiology

Researchers began studying biological rhythms approximately 50 years ago. Although no single experiment serves as the defining event from which to date the beginning of modern research in chronobiology, studies conducted in the 1950s on circadian rhythmicity in fruit flies by Colin Pittendrigh and in humans by Jürgen Aschoff can be considered its foundation. The area of sleep research, which also is subsumed under the field of chronobiology, evolved somewhat independently, with the identification of various sleep stages by Nathaniel Kleitman around the same time (Dement 2000). The legacies of these pioneers continue today with the advancement of the fields they founded.

The roots of the study of biological rhythms, however, reach back even further, to the 1700s and the work of the French scientist de Mairan, who published a monograph describing the daily leaf movements of a plant. De Mairan observed that the daily raising and lowering of the leaves continued even when the plant was placed in an interior room and thus was not exposed to sunlight. This finding suggested that the movements represented something more than a simple response to the sun and were controlled by an internal clock.

## Characteristic Properties of Circadian Rhythms

De Mairan’s apt observations illustrate one critical feature of circadian rhythms— their self-sustained nature. Thus, almost all diurnal rhythms that occur under natural conditions continue to cycle under laboratory conditions devoid of any external time-giving cues from the physical environment (e.g., under constant light or constant darkness). Circadian rhythms that are expressed in the absence of any 24-hour signals from the external environment are called free running. This means that the rhythm is not synchronized by any cyclic change in the physical environment. Strictly speaking, a diurnal rhythm should not be called circadian until it has been shown to persist under constant environmental conditions and thereby can be distinguished from those rhythms that are simply a response to 24-hour environmental changes. For practical purposes, however, there is little reason to distinguish between diurnal and circadian rhythms, because almost all diurnal rhythms are found to be circadian. Nor is a terminology distinction made among circadian rhythms based on the type of environmental stimulus that synchronizes the cycle.

The persistence of rhythms in the absence of a dark-light cycle or other exogenous time signal (i.e., a Zeitgeber) clearly seems to indicate the existence of some kind of internal timekeeping mechanism, or biological clock. However, some investigators have pointed out that the persistence of rhythmicity does not necessarily exclude the possibility that other, uncontrolled cycles generated by the Earth’s revolution on its axis might be driving the rhythm (see Aschoff 1960).

The hypothesis that such uncontrolled geomagnetic cues might play a role in the persistence of rhythmicity can be refuted by a second characteristic feature of circadian rhythms: These cycles persist with a period of close to, but not exactly, 24 hours. If the rhythms were exogenously driven, they should persist with a period of exactly 24 hours. The seeming imprecision is an important feature of rhythmicity, however. As Pittendrigh (1960) demonstrated, the deviation from a 24-hour cycle actually provides a means for the internal timekeeping system to be continuously aligned by and aligned to the light-dark environment. This continuous adjustment results in greater precision in controlling the timing, or phase, of the expressed rhythms, because little drift is allowed to occur before the rhythm is “reset” to the correct phase.

A third characteristic property of circadian rhythms is their ability to be synchronized, or entrained, by external time cues, such as the light-dark cycle. Thus, although circadian rhythms can persist in the absence of external time cues (meaning that they are not driven by the environment), normally such cues are present and the rhythms are aligned to them. Accordingly, if a shift in external cues occurs (e.g., following travel across time zones), the rhythms will be aligned to the new cues. This alignment is called entrainment.

**Figure 1:**
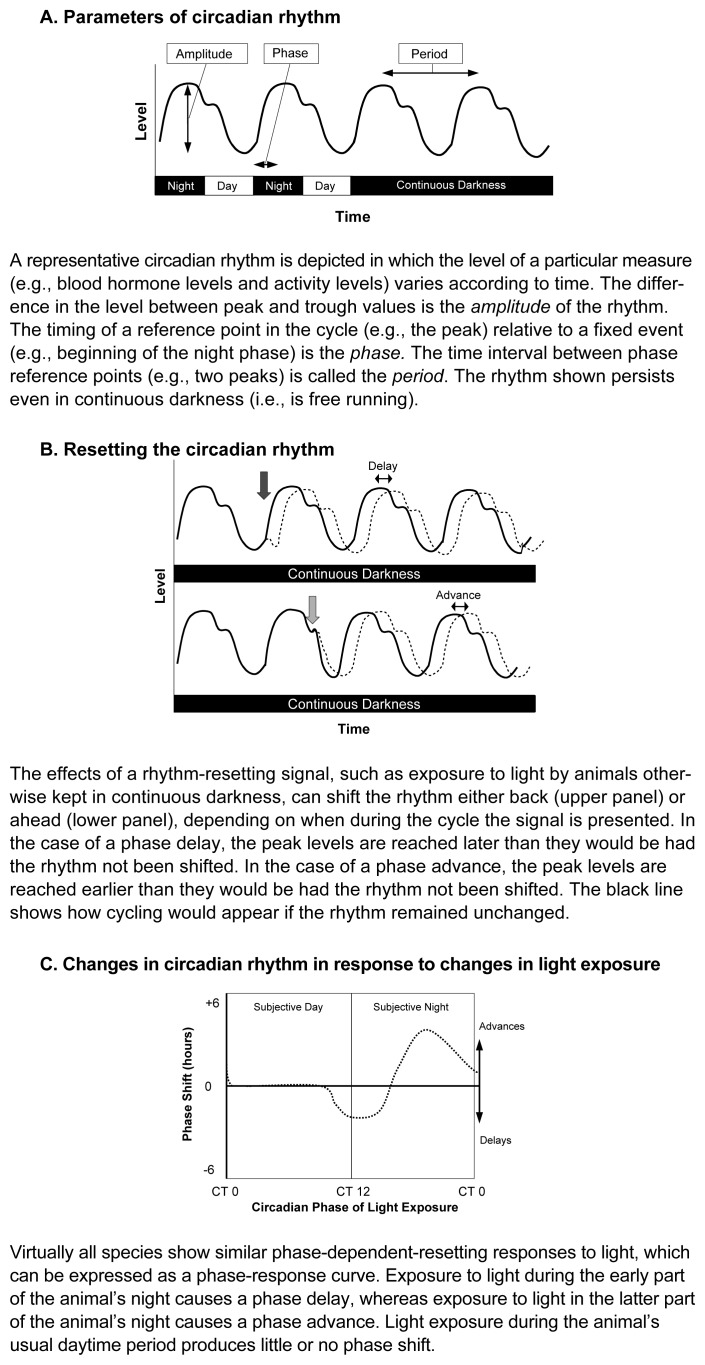
Circadian rhythm responses to light.

Initially, it was unclear whether entrainment was achieved by modulating the rate of cycling (i.e., whether the cycle was shortened or lengthened until it was aligned to the new cues and then reverted to its original length) or whether entrainment was achieved by discrete “resetting” events. Experiments resulting from this debate led to fundamental discoveries. For example, researchers discovered that the organism’s response to light (i.e., whether a cycle advances, is delayed, or remains unchanged) differs depending on the phase in the cycle at which it is presented (Pittendrigh 1960). Thus, exposure to light during the early part of the individual’s “normal” dark period generally results in a phase delay, whereas exposure to light during the late part of the individual’s normal dark period generally results in a phase advance. This difference in responses can be represented by a phase-response curve (see figure 1 for a schematic illustration of a circadian cycle as well as a phase-response curve). Such a curve can predict the manner in which an organism will entrain not only to shifts in the light-dark cycles but also to unusual light cycles, such as non-24-hour cycles or different light:dark ratios. The existence of a phase-response curve also implies that entrainment is achieved by discrete resetting events rather than changes in the rate of cycling.

In addition to the timing of the light exposure, the light intensity can modulate cycling periods when organisms are left in constant light. Thus, exposure to brighter light intensities can lengthen the period in some species and shorten it in other species. This phenomenon has been dubbed “Aschoff’s rule” (Aschoff 1960). Ultimately, both mechanisms of entrainment appear to be aspects of the same thing, because the consequences of Aschoff’s rule can be predicted or explained by the phase-response curves to light.

Although the light-dark cycle clearly is the major Zeitgeber for all organisms, other factors—such as social interactions, activity or exercise, and even temperature—also can modulate a cycle’s phase. The influence of temperature on circadian rhythms is particularly interesting in that a change in temperature can affect the phase of a cycle without substantially altering the rate of cycling. This means that the cycle may start at an earlier or later-than-normal time but still have the same length. On the one hand, this ability of the internal clock’s pacemaker to compensate for changes in temperature is critical to its ability to predict and adapt to environmental changes, because a clock that speeds up and slows down as the temperature changes would not be useful. On the other hand, temperature compensation also is rather puzzling, because most kinds of biological processes (e.g., biochemical reactions in the body) are accelerated or slowed by temperature changes. Ultimately, this riddle has provided a clue to the nature of the internal clock— that is, the fact that circadian rhythms have a genetic basis. Such a program of gene expression would be more resistant to temperature alteration than, for example, a simple biochemical reaction.

Two final properties of circadian rhythms also provide important hints of the rhythms’ makeup. One of these properties is the rhythms’ ubiquity in nature: Circadian rhythms exist in a broad array of biological processes and organisms, with similar properties and even similar phase-response curves to light. The other property is that circadian rhythms appear to be generated at the cellular level, because the rhythms of unicellular organisms (e.g., algae or the dinoflagellate *Gonyaulax*) are much the same as rhythms of highly complex mammals. Both of these observations suggest that a cycle in the activation (i.e., expression) of certain genes might underlie the timekeeping mechanism.

## The Anatomical Organization of the Internal Clock

Although studies of unicellular organisms point to the cellular nature of the system generating circadian rhythms, the circadian pacemaker in higher organisms is located in cells of specific structures of the organism. These structures include certain regions of the brain (i.e., the optic and cerebral lobes) in insects; the eyes in certain invertebrates and vertebrates; and the pineal gland, which is located within the brain, in nonmammalian vertebrates. In mammals, the circadian clock resides in two clusters of nerve cells called the suprachiasmatic nuclei (SCN), which are located in a region at the base of the brain called the anterior hypothalamus.

The role of the SCN was demonstrated by the landmark discovery in the early 1970s that by damaging (i.e., lesioning) the SCN in rats, researchers could disrupt and abolish endocrine and behavioral circadian rhythms (for a review, see Klein et al. 1991). Furthermore, by transplanting the SCN from other animals into the animals with the lesioned SCN, investigators could restore some of the circadian rhythms. Finally, the SCN’s role as a master pacemaker regulating other rhythmic systems was confirmed by similar studies in hamsters, which demonstrated that the restored rhythms exhibited the clock properties (i.e., the period, or phase, of the rhythm) of the donor rather than of the host (Ralph et al. 1990). The discovery that the SCN is the site of primary regulation of circadian rhythmicity in mammals gave researchers a focal point for their research: if one wanted to understand 24-hour timekeeping, one needed to study the clock in the SCN.

Recently, however, researchers have been surprised to find that circadian rhythms could persist in isolated lungs, livers, and other tissues grown in a culture dish (i.e., in vitro) that were not under the control of the SCN (Yamazaki et al. 2000). These observations indicate that most cells and tissues of the body may be capable of modulating their activity on a circadian basis. Such findings do not, however, diminish the central role played by the SCN as the master circadian pacemaker that somehow coordinates the entire 24-hour temporal organization of cells, tissues, and the whole organism. The physiological mechanisms underlying this coordination include signals emitted by the SCN that act on other nerve cells (i.e., neural signals) or which are also distributed through the blood to other organs (i.e., neurohormonal signals). To date, however, the characteristics of the circadian signal itself—that is, the specific manner in which the SCN “talks” to the rest of the body—remain unknown (see Stokkan et al. 2001).

Although the effects of SCN lesions on numerous rhythms have been elucidated, their effects on sleep are less clear. Thus, SCN lesions clearly disrupt the consolidation and pattern of sleep in rats but have only minimal effects on the animals’ amount of sleep or sleep need (Mistlberger et al. 1987). For this and other reasons, researchers have postulated that sleep is subject to two essentially independent control mechanisms: (1) the circadian clock that modulates the propensity for sleep and (2) a homeostatic control that reflects the duration of prior waking (i.e., “sleep debt”). Recently, however, studies in squirrel monkeys found that SCN lesions can affect the amount of sleep. Moreover, sleep studies in mice carrying changes (i.e., mutations) in two of the genes influencing circadian cycles (i.e., the *DBP* and *Clock* genes) indicated that these mutations resulted in changes in sleep regulation (Naylor et al. 2000; Franken et al. 2000). Both of these observations raise the intriguing possibility that the homeostatic and circadian controls may be more interrelated than researchers previously thought.

## Molecular Genetics of Circadian Rhythms

As discussed previously, the properties of circadian clocks suggested cyclic changes in the expression of certain genes as a possible mechanism underlying the internal pacemaker. This hypothesis was supported by the demonstration in a number of species that the expression of genes and the production of proteins encoded by those genes were required for normal clock function. Nevertheless, a completely different experimental approach ultimately led to the identification of molecular circadian clock components. Researchers used chemical agents to introduce numerous, random mutations into the DNAs of the fruit fly, *Drosophila melanogaster*, and of the filamentous fungus *Neurospora*. The resulting mutant organisms then were screened for rhythm abnormalities. This mutagenesis approach led to the identification of the first circadian clock mutants, which were called *period* (*per*) and *frequency* (*frq*, pronounced “freak”). The genes that carried the mutations in these organisms were cloned in the 1980s (for a review, see Wager-Smith and Kay 2000). However, considerable frustration ensued as researchers sought to isolate the equivalent genes in mammals (i.e., mammalian homologs). Finally, in the early 1990s, researchers began a similar mutagenesis screening approach in the mouse and described the first mouse circadian mutation, called *Clock*, in 1994 (see King and Takahashi 2000). In 1997 the gene affected by this mutation became the first mammalian circadian clock gene to be cloned (King and Takahashi 2000). Like the mutants of the *Per* and *Frq* genes, the altered *Clock* gene both affected the free-running rhythm period (i.e., lengthened the period) and caused a loss of persistence of circadian rhythms under constant environmental conditions. Both the C*lock* mutant in mice and the *Per* mutant in flies were the first animals of their respective species identified using such a mutagenesis approach in which the mutation manifested as altered behavior rather than an altered physiological process.

**Figure 2:**
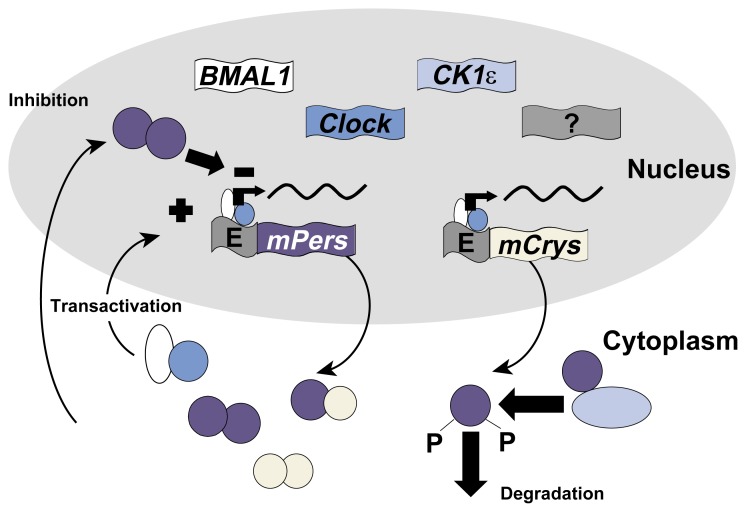
Schematic representation of the regulation of genes believed to be involved in the circadian clock. *BMAL1, Clock, CK1*ɛ, *mPer*, and *mCry* all are circadian clock genes identified in mice. (Several variants exist of the *mPer* and *mCry* genes.) In the cell’s nucleus, the genetic information encoded in these genes is converted into a carrier molecule called mRNA (black wavy lines), which is transported into the fluid within the cell (i.e., the cytoplasm). There, the mRNA is used to generate the protein products encoded by the circadian clock genes (circles and ovals with colors corresponding to the respective genes). Some of these proteins regulate the activity of certain clock genes by binding to “molecular switches” (i.e., E boxes) located in front of those genes. This is called a feedback cycle. Thus, the BMAL1 and clock proteins promote activation of the *Per* and *mCry* genes, whereas Per proteins inhibit activation of those genes. The 24-hour cycling comes about as the BMAL1 and *Clock* proteins induce increased production of *Per* and *Cry* proteins. As *Pers* and *Crys* accumulate, they inhibit their own synthesis, and the protein levels decline. CK1ɛ protein also helps to regulate *Clock* protein levels by destabilizing *Per* protein. NOTE: BMAL1 = brain and muscle ARNT-like 1; CK1ɛ = caseine kinase 1 epsilon; mPer = mouse period; mCry = mouse cryptochrome.

Since the discovery of the *Clock* gene in mice, the list of circadian clock genes identified in mammals has grown in a remarkably short period of time (see table 1). For example, researchers have identified not one, but three mammalian genes that correspond to the *per* gene in both their structure (i.e., nucleotide sequence) and their function (King and Takahashi 2000; Lowrey and Takahashi 2000). Some of the proposed circadian clock genes have been identified solely based on their similarity in sequence to *Drosophila* clock genes and have not been confirmed to have clock function based on an examination of the behavior of the corresponding mutants. Nevertheless, the findings to date clearly indicate the outline of a pacemaker that is based on a feedback cycle of gene expression (see figure 2).

## Importance of the Circadian Clock for Human Health and Well-Being

Nearly all physiological and behavioral functions in humans occur on a rhythmic basis, which in turn leads to dramatic diurnal rhythms in human performance capabilities. Regardless of whether it results from voluntary (e.g., shift work or rapid travel across time zones) or involuntary (e.g., illness or advanced age) circumstances, a disturbed circadian rhythmicity in humans has been associated with a variety of mental and physical disorders and may negatively impact safety, performance, and productivity. Many adverse effects of disrupted circadian rhythmicity may, in fact, be linked to disturbances in the sleep-wake cycle. Some rhythmic processes are more affected by the circadian clock than by the sleep-wake state, whereas other rhythms are more dependent on the sleep-wake state.

For most animals, the timing of sleep and wakefulness under natural conditions is in synchrony with the circadian control of the sleep cycle and all other circadian-controlled rhythms. Humans, however, have the unique ability to cognitively override their internal biological clock and its rhythmic outputs. When the sleep-wake cycle is out of phase with the rhythms that are controlled by the circadian clock (e.g., during shift work or rapid travel across time zones), adverse effects may ensue.

In addition to the sleep disturbances associated with jet lag or shift work, sleep disorders can occur for many other known and unknown reasons. And although disturbed sleep is a hallmark of many human mental and physiological disorders, notably affective disorders, it is often unclear whether the sleep disturbances contribute to or result from the illness. Other circadian rhythm abnormalities also are often associated with various disease states, although again the importance of these rhythm abnormalities in the development (i.e., etiology) of the disease remains unknown (Brunello et al. 2000).

One important factor contributing to researchers’ inability to precisely define the role of circadian abnormalities in various disease states may be the lack of knowledge of how circadian signals from the SCN are relayed to target tissues. To further elucidate the regulation of circadian rhythms, researchers need a better understanding of the nature of circadian signal output from the SCN and of how these output signals may be modified once they reach their target systems. Such an enhanced understanding also would allow for a better delineation of the importance of normal temporal organization for human health and disease. The finding that two major causes of death—heart attacks and strokes— show time-of-day variation in their occurrence is a case in point. If scientists knew more about the mechanisms responsible for the rhythmicity of these disorders, they might be able to identify more rational therapeutic strategies to influence these events. Finally, given that dramatic changes occur in the circadian clock system with advanced age, these changes may underlie, or at least exacerbate, the age-related deterioration in the physical and mental capabilities of older adults.

## Conclusions

Although researchers in just the past few years have made great advances in understanding the molecular basis of circadian rhythmicity, this progress builds on extensive research carried out in many laboratories during the past 50 years. Within the same period, other researchers in numerous laboratories have elucidated the critical role played by the SCN in the regulation of circadian rhythmicity in mammals and perhaps other vertebrates. (For more information on these findings and their relevance, the reader can refer to a variety of resources on the World Wide Web, some of which are listed in table 2.)

Most animals are content to obey their SCN and let it orchestrate the expression of a multitude of circadian rhythms. Humans, however, have a mind of their own and often use this mind to disobey their “internal clock”—for example, with an increasing tendency toward 24-hour availability for business. The potential consequences of such an increasingly 24-hour on-call lifestyle are unknown at this point, but the evidence does not bode well.

The challenge for researchers and clinicians now is to determine not only the cause but also the consequences for human health and disease of disruptions in the temporal organization of the circadian system. These issues also include the question of what role alcohol may play in the disruption of normal circadian rhythms and the biological clock. This question is addressed in more detail in this special issue of *Alcohol Research & Health*. Drs. Wasielewski and Holloway review ways in which alcohol and the body’s circadian rhythm interact, using body temperature as an index of circadian rhythm function. The sleep-wake cycle, which constitutes a central aspect of circadian rhythms in particular, is subject to modification by alcohol; alcohol’s effects on the sleep of nonalcoholics and alcoholics are discussed by Drs. Roehrs and Roth and by Dr. Brower, respectively.

As indicated in this article, disturbances of the normal circadian rhythmicity can result in serious health consequences, including psychiatric disorders, such as depression. At the same time, psychoactive drugs, such as antidepressants, also have chronobiological effects. Dr. Rosenwasser explores those associations and discusses alcohol’s effects in human and animal models of depression. Other influences of alcohol on the biological clock may be even more subtle and remain rather speculative, such as the consequences of prenatal alcohol exposure, which is discussed by Drs. Earnest, Chen, and West. Finally, not only may alcohol consumption affect circadian rhythms, but circadian factors, such as the light-dark cycle, may also influence alcohol consumption. This topic is discussed by Drs. Hiller-Sturmhöfel and Kulkosky. Together, these articles offer readers insight into the interesting and complex interactions that exist between alcohol and the circadian rhythms that govern much of the behavior and well-being of all organisms, including humans.

## Figures and Tables

**Table 1:** Mammalian Circadian Clock Genes; the Corresponding Genes in the Fruit Fly, *Drosophila*; and the Effects of Changes (i.e., Mutations) in Those Genes on the Behavior (i.e., Phenotype) of the Affected Animals

Mouse Gene	Alias	*Drosophila* Gene	Mutant Phenotype
*Clock		*dClock*	Lengthened period; loss of persistent rhythmicity in constant conditions
*mPer1*		*period*	Reduced amplitude, shortened period, or loss of rhythm
*mPer2		*period*	Shortened period, loss of rhythm
*mPer3		*period*	Modest shortening of period
*CKIɛ	*tau (hamster)*	*doubletime*	Shortened period in hamster mutants
*mCry1 *mCry2*		*dcry*	Animals lacking the *mCry1* gene (i.e., *mCry1* knockouts) have shortened period; *mCry2* knockouts have lengthened period; animals lacking both genes (i.e., double knockouts) have a loss of rhythm
*BMAL1	*MOP3*	*cycle*	Loss of rhythm
?*mTim*		*timeless*	Role in mammals is not clear
?*DBP*			Modest lengthening of period

NOTE: Asterisk (*) indicates that a key role for the gene in timekeeping has been demonstrated by the phenotype of a mutant.

**Table 2:** Chronobiological Resources on the World Wide Web

Web Site	Description
http://www.nwu.edu/ccbm/	Web site of Northwestern University’s Center for Sleep and Circadian Biology
http://www.sleepquest.com/	Information site of William Dement’s Sleep Research Center
http://www.med.stanford.edu/school/Psychiatry/narcolepsy	Narcolepsy site created by Emmanuel Mignot at Stanford University
http://www.sleepfoundation.org/	Web site of the National Sleep Foundation
http://www.srbr.org/	Web site of the Society for Research on Biological Rhythms
http://www.cbt.virginia.edu/	Web site of the Center for Biological Timing at the University of Virginia
http://www.hhmi.org/grants/lectures	Web site providing Howard Hughes Medical Institute Holiday Lectures

## Footnotes

1 For a definition of this and other technical terms used in this article and throughout this issue of the journal, please see glossary, p. 92.

## GLOSSARY

*Every scientific field has its specific terminology; the scientific area of biological rhythms and sleep is no exception. This glossary defines some of the terms that readers may encounter in this article and throughout this special issue of* Alcohol Research & Health.

Chronobiology: A subdiscipline of biology concerned with the timing of biological events, especially repetitive or cyclical phenomena, in individual organisms.
Circadian: A term derived from the Latin phrase “circa diem,” meaning “about a day”; refers to biological variations or rhythms with a cycle of approximately 24 hours. Circadian rhythms are self-sustaining (i.e., *free running*), meaning that they will persist when the organism is placed in an environment devoid of time cues, such as constant light or constant darkness. For comparison, see *diurnal, infradian*, and *ultradian*.
Circadian time (CT): A standardized 24-hour notation of the phase in a circadian cycle that represents an estimation of the organism’s subjective time. CT 0 indicates the beginning of a subjective day, and CT 12 is the beginning of a subjective night. For example, for a nocturnal rodent, the beginning of a subjective night (i.e., CT 12) begins with the onset of activity, whereas for a diurnal species, CT 0 would be the beginning of activity. For comparison, see *Zeitgeber time*.
DD: A conventional notation for an environment kept in continuous darkness (as opposed to a light-dark cycle). For comparison, see *LD*.
Diurnal: Varying with time of day. Diurnal rhythms may persist when the organism is placed in an environment devoid of time cues, such as constant light or constant darkness. Therefore, diurnal variations can be either light driven or clock driven. For comparison, see *circadian*.
Entrainment: The process of synchronization of a timekeeping mechanism to the environment, such as to a light-dark cycle, or LD. For comparison, see *free running*.
Free running: The state of an organism (or rhythm) in the absence of any entraining stimuli. Typically, subjects are kept in constant dim light or constant darkness to assess their free-running rhythms. For comparison, see *entrainment*.
Infradian: A term derived from the Latin phrase “infra diem,” meaning “less than a day”; refers to biological cycles that last more than 1 day and, therefore, have a frequency of less than one per day. For comparison, see *circadian* and *ultradian*.
LD: Conventional notation for a light-dark environmental cycle; the numbers of hours of light and dark are typically presented separated by a colon. For example, LD 16:8 denotes a cycle consisting of 16 hours of light and 8 hours of dark. For comparison, see *DD*.
Masking: The obscuring of the “true” state of a rhythm by conditions that prevent its usual expression. Usually, the phase of an entrained rhythm or the absence of *entrainment* (e.g., in an animal that is unable to entrain because of some defect) is said to be masked by a light cycle. For example, the aversion of a nocturnal rodent to bright light results in its activity onset appearing to coincide with the absence of light, or “lights off,” when the animal actually has been awake for hours. For comparison, see *entrainment*.
Nonrapid eye movement (NREM) sleep: Sleep stages that include the “deeper” stages of sleep in which dreaming typically does not occur. Also referred to as slow-wave sleep. For comparison, see *rapid eye movement sleep*.
Phase shift: A change in the phase of a rhythm. This change can be measured by observing a change in the timing of a phase reference point (e.g., activity onset or the nocturnal rise in the release of the hormone melatonin) from the timing expected based on previous, *free-running* cycles. Phase shifts may be either advances (i.e., the phase reference point occurs earlier than normal) or delays (i.e., the phase reference point occurs later than normal).
Phase-response curve (PRC): A graphical summary of the *phase shifts* produced by a particular manipulation, such as a light pulse or a pharmacological treatment, as a function of the phase (i.e., *circadian time*) at which the manipulation occurs. Defining the PRC to light has enabled researchers to understand and predict how *entrainment* to light cycles is accomplished.
Rapid eye movement (REM) sleep: A stage of light sleep characterized by rapid eye movements and associated with dreaming. Also called paradoxical sleep. For comparison, see *nonrapid eye movement sleep*.
Suprachiasmatic nucleus or nuclei (SCN): A cluster of nerve cells located in the brain region called the hypothalamus that is responsible for generating and coordinating *circadian* rhythmicity in mammals.
Ultradian: A term derived from the Latin phrase “ultra diem,” meaning “more than a day”; refers to biological cycles that last less than 1 day and, therefore, have a frequency of more than one per day. For comparison, see *circadian* and *infradian*.
Zeitgeber: A German word literally meaning “time-giver.” A time cue capable of entraining *circadian* rhythms. Light represents the most important Zeitgeber.
Zeitgeber time (ZT): A standardized 24-hour notation of the phase in an entrained *circadian* cycle in which ZT 0 indicates the beginning of day, or the light phase, and ZT 12 is the beginning of night, or the dark phase. For comparison, see *circadian time*.
